# Bridging Technology and Pretest Genetic Services: Quantitative Study of Chatbot Interaction Patterns, User Characteristics, and Genetic Testing Decisions

**DOI:** 10.2196/73391

**Published:** 2025-09-17

**Authors:** Yang Yi, Lauren Kaiser-Jackson, Jemar R Bather, Melody S Goodman, Daniel Chavez-Yenter, Richard L Bradshaw, Rachelle Lorenz Chambers, Whitney F Espinel, Rachel Hess, Devin M Mann, Rachel Monahan, David W Wetter, Ophira Ginsburg, Meenakshi Sigireddi, Kensaku Kawamoto, Guilherme Del Fiol, Saundra S Buys, Kimberly A Kaphingst

**Affiliations:** 1 Department of Communication University of Utah Salt Lake City, UT United States; 2 Huntsman Cancer Institute Salt Lake City, UT United States; 3 Center for Anti-Racism, Social Justice & Public Health School of Global Public Health New York University New York United States; 4 Department of Biostatistics School of Global Public Health New York University New York, NY United States; 5 Division of Hematology-Oncology Perelman School of Medicine University of Pennsylvania Philadelphia United States; 6 Department of Medical Ethics and Health Policy Perelman School of Medicine University of Pennsylvania Philadelphia, PA United States; 7 Department of Biomedical Informatics Spencer Fox Eccles School of Medicine University of Utah Salt Lake City, UT United States; 8 Perlmutter Cancer Center NYU Langone Health New York, NY United States; 9 Department of Population Health Sciences Spencer Fox Eccles School of Medicine University of Utah Salt Lake City, UT United States; 10 Department of Internal Medicine Spencer Fox Eccles School of Medicine University of Utah Salt Lake City, UT United States; 11 Department of Population Health NYU Grossman School of Medicine New York City, NY United States; 12 Center for Health Outcomes and Population Equity (HOPE) Huntsman Cancer Institute Salt Lake City, UT United States; 13 Center for Global Health National Cancer Institute Rockville, MD United States

**Keywords:** cancer genetics, chatbot delivery, user interaction, genetic testing, pretest genetic services

## Abstract

**Background:**

Among the alternative solutions being tested to improve access to genetic services, chatbots (or conversational agents) are being increasingly used for service delivery. Despite the growing number of studies on the accessibility and feasibility of chatbot genetic service delivery, limited attention has been paid to user interactions with chatbots in a real-world health care context.

**Objective:**

We examined users’ interaction patterns with a pretest cancer genetics education chatbot as well as the associations between users’ clinical and sociodemographic characteristics, chatbot interaction patterns, and genetic testing decisions.

**Methods:**

We analyzed data from the experimental arm of Broadening the Reach, Impact, and Delivery of Genetic Services, a multisite genetic services pragmatic trial in which participants eligible for hereditary cancer genetic testing based on family history were randomized to receive a chatbot intervention or standard care. In the experimental chatbot arm, participants were offered access to core educational content delivered by the chatbot with the option to select up to 9 supplementary informational prompts and ask open-ended questions. We computed descriptive statistics for the following interaction patterns: prompt selections, open-ended questions, completion status, dropout points, and postchat decisions regarding genetic testing. Logistic regression models were used to examine the relationships between clinical and sociodemographic factors and chatbot interaction variables, examining how these factors affected genetic testing decisions.

**Results:**

Of the 468 participants who initiated a chat, 391 (83.5%) completed it, with 315 (80.6%) of the completers expressing a willingness to pursue genetic testing. Of the 391 completers, 336 (85.9%) selected at least one informational prompt, 41 (10.5%) asked open-ended questions, and 3 (0.8%) opted for extra examples of risk information. Of the 77 noncompleters, 57 (74%) dropped out before accessing any informational content. Interaction patterns were not associated with clinical and sociodemographic factors except for prompt selection (varied by study site) and completion status (varied by family cancer history type). Participants who selected ≥3 prompts (odds ratio 0.33, 95% CI 0.12-0.91; *P*=.03) or asked open-ended questions (odds ratio 0.46, 95% CI 0.22-0.96; *P*=.04) were less likely to opt for genetic testing.

**Conclusions:**

Findings highlight the chatbot’s effectiveness in engaging users and its high acceptability, with most participants completing the chat, opting for additional information, and showing a high willingness to pursue genetic testing. Sociodemographic factors were not associated with interaction patterns, potentially indicating the chatbot’s scalability across diverse populations provided they have internet access. Future efforts should address the concerns of users with high information needs and integrate them into chatbot design to better support informed genetic decision-making.

## Introduction

### Background

Access to cancer genetic services is a crucial factor for cancer prevention and education, especially for those with familial cancer predisposition [1]. Demand for cancer genetic services is increasing due to technological and genomic science advances [2,3]. In the traditional genetic service delivery model, patients meet with a certified genetic counselor through a referral for a pretest genetic counseling appointment to discuss details of genetic testing (eg, genetics basics and testing options and procedures), allowing patients to make an informed decision about testing [3,4]. Although cancer genetic services are vital, many patients who meet eligibility criteria do not receive them [5,6]. Historically marginalized communities especially face hurdles such as limited resources and inadequate referrals, restricting essential testing and care [6,7]. In addition, the traditional model can be time-consuming and difficult to schedule in the face of workforce shortages [3]. To improve access, alternative models of service delivery have been developed and tested [8-12]. These approaches include telehealth appointments [13], use of genetic counseling assistants to optimize genetic counselor time [14], video- or web-based education [15], and digital health tools such as chatbots [16]. Each of these alternative delivery models has its own unique attributes as well as distinct advantages and disadvantages compared to traditional in-person genetic counseling [3].

Among the alternative solutions, chatbots (or conversational agents) are being increasingly used for service delivery amid the current technological evolution [17]. Chatbots are automated, programmed agents designed to simulate human interactions. They can use natural language processing to interpret user inputs and provide appropriate responses in human language through text or voice [18]. Chatbots have been adopted in many health care contexts, such as health behavior change interventions for smoking cessation and substance use reduction [19,20], promotion of health habits such as daily physical activity and stair-climbing habits among office workers [21,22], and addressing mental health concerns such as psychological distress and stress [23,24]. Specific to remote health service delivery, the 2024 review by Laymouna et al [17] summarized health care chatbot roles under 3 main types: patient support and care management, education and skill building, and health behavior promotion. This study focused on chatbots in delivering cancer genetic pretest education and expanding access to genetic testing.

Recent research has explored how chatbots can be effective remote service delivery alternatives for cancer genetic services [25]. Chatbots can play a vital role in risk screening and assessment based on individuals’ family history, as well as providing pretest education and counseling for patients considering genetic testing [25-27]. Previous studies have shown the acceptability and feasibility of using chatbots for these purposes [26,27]. They have particularly noted that participants favor chatbots for tasks of moderate complexity, viewing them as a useful complement, rather than a replacement, to standard care [28]. The Broadening the Reach, Impact, and Delivery of Genetic Services (BRIDGE) randomized controlled trial showed that use of pretest cancer genetic services and completion of genetic testing were statistically equivalent between chatbot and standard-of-care service delivery models [29]. Previous studies have evaluated user interactive experiences with chatbots via a qualitative approach, such as through focus groups [30] and interviews [31]. Several survey studies targeting chatbot usability evaluations have included measures of user interactive experiences and service satisfaction within the surveys [26,32]. Notably, despite the growing number of studies on the accessibility and feasibility of chatbot health service delivery, limited attention has been paid to user interactions with chatbots in a real-world health care context—specifically, the reciprocal actions toward chatbot content [33]. However, valuable insights can be gained on user-chatbot interactions, such as identifying which prompts lead users to seek more information when they drop out of a conversation and the specific open-ended questions they ask. Gathering these data can guide future chatbot designs, making them more responsive to user needs. To address this gap, Chavez-Yenter et al [33] studied user interactions with the BRIDGE chatbot for pretest genetic education in a small feasibility study. Of 36 who started the chat, 30 completed it—most completers (21/30, 70%) showed interest in pursuing genetic testing, whereas the rest were uncertain. Participants who decided to take the test selected an average of 1.87 (SD 1.2) informational prompts and rarely asked open-ended questions, whereas participants who were unsure about taking the test selected an average of 3.67 (SD 2.9) prompts and typically asked at least one open-ended question [33]. Our study expands on prior work by examining the interactions of a large sample of users randomized to the arm receiving the pretest genetics education chatbot in the BRIDGE randomized controlled trial. This trial examined user interactions among primary care patients across 2 major US health care systems. In total, 3073 patients took part in the trial, including 1554 assigned to the experimental arm, all of whom were invited to use the chatbot.

Unlike the aforementioned descriptive feasibility study, our study also explored the potential influence of clinical and sociodemographic factors on use patterns. Understanding whether clinical factors such as inherited predisposition to certain cancers or having a primary care physician affect user interaction patterns can help tailor future chatbots to improve accessibility and interactivity, better addressing the needs of individuals with different clinical backgrounds [33,34]. To effectively leverage chatbots in expanding health care services and reducing inequities, it is crucial to enhance user experience across diverse population groups [17]. Key demographic factors such as age, sex and gender, race and ethnicity, and geographic location must be carefully considered to ensure equitable and inclusive service delivery. For instance, previous research in cancer genetics has demonstrated that, even when traditional barriers to genetic testing such as cost, insurance, and access are minimized, minoritized racial-ethnic patients remain less likely to receive recommended genetic care [35]. Gender-based differences in genetic testing awareness were highlighted in the focus group study by Hamilton et al [36]—male participants demonstrated a broader understanding of various disease contexts, including the use of genetic testing for paternity determination, whereas female participants primarily focused on their experiences with prenatal testing and reproductive health. Although not cancer-specific, the systematic review by Best et al [37] found that rural populations faced greater shortages in genetic resources than urban communities. This important issue needs investigation for chatbot delivery models. The 2023 review by Webster et al [25] of chatbot use in genetic cancer risk assessment and counseling found that only 2 out of 7 studies provided detailed data on patient demographics such as age, race, ethnicity, and sex. The 2021 study by Nazareth et al [27] revealed that 58.8% of the 61,070 genetic service chatbot users identified as non-Hispanic White, with users being significantly younger than nonusers [27]. The 2021 study by Heald et al [26] of 506 patients accessing a genetic service chatbot showed that 86% were White and 96% were non-Hispanic. It also found that individuals who were female, White, and received the chatbot link via a patient portal were more likely to engage with the chatbot, whereas Black individuals were less likely to do so [26]. The aforementioned insights show that genetic service delivery models work differently across various populations, highlighting the need to examine these differences further. In the context of chatbot-based delivery, Laymouna et al [17] found that chatbots can effectively serve diverse groups regardless of age, gender, race, or socioeconomic status but cautioned against replacing established health services given the potential to widen health disparities. Accordingly, our study explored whether certain groups may perceive the chatbot’s design or content more favorably. This underscores the importance of investigating how user interactions vary according to sociodemographic factors.

Overall, due to the limited research on how patients use cancer genetic service chatbots—particularly in large-scale, real-world health care settings—and the lack of empirical evidence on how clinical and sociodemographic factors influence those use patterns, this study was conducted to fill those gaps.

### Objectives

To address these research gaps, this study explored the use patterns of a pretest genetics education chatbot in 2 large health care systems. The primary outcomes were to examine participants’ core interactive experiences: user interactions with informational prompts (how many times they opted to view more informational content), user open-ended questions (how many questions they asked and of what nature), and genetic testing decision after the completion of the chat. In addition, to better capture the nuances of user interactive actions, we explored 2 secondary outcomes, that is, user conversation completion status (whether an individual completed the entire content of the pretest education) and user dropout points (for participants who did not complete the entire chat, whether a user left the chat before any informational content). Furthermore, given the intention of using an educational chatbot to increase access to genetic counseling services for hereditary cancer, it is pertinent to explore whether clinical and sociodemographic factors are related to differences in the participants’ interaction with the educational content on genetics, as well as their genetic testing intention. Analyzing how patient characteristics related to their use of the chatbot, as well as their genetic testing decision after completing the chat, is important for a better understanding and development of future genetics education chatbots.

## Methods

### Study Design

#### Procedures

The BRIDGE randomized controlled trial has been previously described [38]. Eligible participants in 2 large health care systems (University of Utah Health [UHealth] and New York University Langone Health [NYULH]) were randomized 1:1 to either chatbot or enhanced standard-of-care genetic service delivery models. The chatbot was designed to deliver automated pretest cancer genetics education to patients who met National Comprehensive Cancer Network guideline-based criteria for cancer genetic testing. The primary aim of BRIDGE was to assess the equivalence of the chatbot delivery model to genetic counseling standard of care with regard to uptake of pretest genetic services and genetic testing [29]. The analysis presented in this paper focused on participants randomized to the intervention arm (n=1554) who were invited to interact with the pretest genetics education chatbot. These participants were sent a patient portal (MyChart in Epic) message inviting them to open the pretest genetics chat via a hyperlink, which launched the chatbot on a web browser independently from their electronic health record (EHR). Nonresponders received a reminder patient portal message 1 week later and up to 2 follow-up telephone calls. For those who started the chat, a transcript of the pretest genetics education chat was used to analyze user interactions in this study.

#### Participants

Patients in the BRIDGE trial were identified as eligible for cancer genetic testing based on their family history of cancer in the EHR [39-43]. Specifically, we used an algorithm-based system to screen family cancer history data at the population level stored in the EHR [44]. The Genetic Cancer Risk Detector algorithm identified unaffected primary care patients at the 2 study sites who met the criteria for genetic evaluation for specific patterns of family history of cancer, suggesting a hereditary syndrome (breast cancer, ovarian cancer, pancreatic cancer, multiple predispositions, or other) [45-47]. Additional eligibility criteria included being aged 25 to 60 years, being English or Spanish speaking, having had a primary care appointment at one of the study sites within the previous 3 years, no previous cancer diagnosis other than nonmelanoma skin cancer, and no previous genetic counseling or testing related to hereditary cancer. Finally, participants had to have an electronic patient portal account or be willing to create one. The inclusion criteria, including cancer family history, were verified by a genetic counseling assistant at each site.

#### Pretest Genetics Chatbot

The research team scripted the rule-based chatbot to reflect the content of a pretest cancer genetic counseling appointment [29,33]. To minimize the impact of users’ digital literacy on their ability to engage with the chatbot, the chatbot’s interface was designed to facilitate navigation through the chat by selecting predetermined responses. The chat began with a recorded video introduction from the lead genetic counselor at each study site. Participants were then asked to answer questions about the number of children and siblings they had, followed by viewing educational content. The educational content was scripted to ensure that all participants viewed a core set of information. They were also presented with 9 decision prompts throughout the chat. Each prompt appeared with 2 or 3 scripted choices that the participant could select, which allowed them to indicate that they would like to see additional information or additional examples for a topic or that they would like to move on. Specifically, the 9 prompts were *genetics basics*, *gene mutations*, *positive result*, *risk percent*, *uncertainty in risk*, *medical record*, *genes included*, *lowering risk*, and *type of results*. There were 4 examples of cancer risk information, including breast cancer as a core example illustrated in the *risk percent* prompt, and the other 3 examples (ie, colon cancer or Lynch syndrome, ovarian cancer, and pancreatic cancer) as optional examples for participants to choose from. After the branching following each prompt, the participant would always return to the core content in the chat script. The chat ended with a final question—“Would you like to move forward with genetic testing?” (response options: “yes,” “no,” and “I’m not sure yet”)—after which the chat was indicated as completed. At any point during the conversation, the participant could type an open-ended question into the chat. The system would use natural language processing to suggest potential questions. The chatbot was trained to provide scripted answers to questions. If the chatbot was unable to answer the question automatically, the question was sent to the study team. Multimedia Appendix 1 provides detailed information on the chatbot script. At any point during the chatbot session, participants had the option to exit the chat and discontinue their participation. For those who did not complete the session, a genetic counseling assistant contacted them up to 3 additional times via MyChart, phone, and mailed letter to answer questions; offer testing; and, in the case of the letter, provide additional information about recommendations based on their family history of cancer.

### Data Collection and Outcomes

#### Overview

Transcripts were coded into a data collection tool in REDCap (Research Electronic Data Capture; Vanderbilt University) [48,49]. Data were extracted from the transcripts and coded by 2 independent coders. Data extracted included time stamps, participant responses, prompt decisions, and open-ended questions and responses.

#### Outcome Measures

Regarding primary outcomes, interactions with informational prompts were counted as the number of times a participant opted to view more informational content. The maximum number of informational prompts was 9. Open-ended questions were counted as the number of questions asked. Genetic testing decision was indicated as *no* (N), *I’m not sure yet* (U), or *yes* (Y).

Regarding secondary outcomes, chat completion status was coded as *did not complete chat* (0) and *completed chat* (1). Dropout point (for those who did not complete the entire chat) was coded as *prior to informational content* (0) and *and after informational content* (1).

Clinical covariates included algorithm criteria met based on specific patterns of family history of cancer suggesting a hereditary syndrome (breast cancer, ovarian cancer, pancreatic cancer, multiple predispositions, or other) and whether the participant had a recorded primary care provider in the EHR (*yes* or *no*).

Regarding sociodemographic covariates, we collected data on age (measured continuously), sex (male or female), race and ethnicity (White, Black, Hispanic, or other), number of children (0 or ≥1) and siblings (0 or ≥1), and preferred language (English or Spanish). We assessed urbanicity (urban or rural) using the 2010 rural-urban commuting area codes and categorized it as urban focused or rural city or town focused [50]. Neighborhood deprivation was measured continuously and assessed using the Neighborhood Deprivation Index [51]. We calculated the Neighborhood Deprivation Index for each zip code provided by our participants using the Housing and Urban Development–US Postal Service ZIP Code Crosswalk file from the 2023 quarter 3 data release [52].

Regarding time of day, we collected participants’ chat start and end times, which were categorized into 5 intervals: early morning (2 AM-10 AM), midday (10 AM-2 PM), afternoon (2 PM-6 PM), evening (6 PM-10 PM), and night (10 PM-2 AM).

#### Statistical Analysis

The data were cleaned and analyzed using SPSS Statistics (version 29.0; IBM Corp). Means and SDs were calculated for continuous variables, and counts and percentages were reported for categorical variables. Descriptive analyses were conducted for participant clinical information, sociodemographic information, and chat interactions. Pearson chi-square tests and independent 2-tailed *t* tests were used to examine differences in variables by study site.

We used logistic regression models to examine associations between participants’ clinical and sociodemographic characteristics and three user interaction outcomes: number of open-ended questions (categorized as *0* vs. *≥1*), conversation completion status, and dropout point. We then used logistic regression models to assess associations between clinical and sociodemographic characteristics and participants’ genetic testing decisions (coded as 0=*No/Not sure yet*; 1=*Yes*). We also evaluated whether user interaction patterns were associated with genetic testing decisions. Therefore, the number of informational prompts and the number of open-ended questions asked were included as predictors. We constructed a multinomial logistic regression model to assess the relationship between clinical and sociodemographic characteristics and the number of informational prompts selected by the participants. On the basis of the distribution, we classified the number of informational prompts selected into 4 dependent variable categories: 0 prompts, 1 prompt, 2 prompts, and ≥3 prompts.

Initially, 481 participants began the pretest education chat. However, 2.8% (13/481) of user interaction transcripts could not be downloaded for log analysis from the web-based portal in the commercial platform due to nonresponsiveness of the commercial platform, resulting in a final analytic sample of 468 participants included in the analysis, with 226 (48.3%) from the NYULH site and 242 (51.7%) from the UHealth site. For the primary outcomes (ie, number of informational prompts, number of open-ended questions asked, and genetic testing decision), we included participants who completed the education chat (391/468, 83.5%) in the analysis, with 47.8% (187/391) from the NYULH site and 52.2% (204/391) from the UHealth site. For the first secondary outcome—completion status—we included all participants who launched the chat (n=468). For the other secondary outcome—dropout point—we included all participants who entered the chat but dropped out during the process (77/468, 16.5%). Due to missing data for race and ethnicity in the EHR (42/468, 9% for those who launched the chat; 34/391, 8.7% for those who completed the chat) in our sample, we conducted analyses on all target outcomes both including and excluding race and ethnicity. We present adjusted odds ratios (ORs) with 95% CIs. Statistical significance was determined at *P*<.05.

### Ethical Considerations

The BRIDGE trial (approval number IRB_00115509) was approved as a single–institutional review board protocol by the University of Utah Institutional Review Board, consistent with current US National Institutes of Health policy for multisite RCTs. Because the trial compared 2 clinical service delivery models, the institutional review board approved a waiver of consent for the procedures described here. Data were de-identified. Participants did not receive compensation for the procedures described here.

## Results

### Overview

#### Participant Characteristics

Table 1 provides detailed information on the 9 informational prompts. Of the 1554 patients randomized to the chatbot arm, 468 (30.12%) started the pretest chat. For participants who opened and started the chat, their mean age was 43.3 (SD 9.5) years, as shown in Table 2. Most participants (n=360, 76.9%) were female, preferred the English language (n=460, 98.3%), identified as White individuals (n=323, 69%), lived in an urban area (n=437, 93.4%), and reported having a primary care provider (n=382, 81.6%).

**Table 1:** Types and content of the 9 informational prompts chosen by chat completers (N=391).

Label	Content	Completers, n (%)
Genetics basics	Provides an overview of genetics and cancer	302 (77.2)
Gene mutations	Includes content explaining gene mutations	6 (1.5)
Positive result	Explains what testing positive for a gene mutation means	10 (2.6)
Risk percent	Provides information on cancer risk percentages for those at increased inherited risk by using breast cancer as an example	4 (1)
Uncertainty in risk	Discusses why there is a range of risk percentages and suggests consulting a genetic counselor to understand personal risk	0 (0)
Medical record	Explains what information in medical records is reviewed to identify a patient as eligible for genetic testing	110 (28.1)
Genes included	Details which genes are included in genetic testing	111 (28.4)
Lowering risk	Provides options to reduce cancer risk	120 (30.7)
Type of results	Provides more explanation of the types of possible genetic testing results, including positive, negative, and variants of uncertain significance	26 (6.6)

**Table 2:** Descriptive statistics of participant sociodemographics by study site.

Characteristic			Overall				NYULH^a^				UHealth^b^				*P* value^c^				
			Participants who started the chat (n=468)		Participants who completed the chat (n=391)		Participants who started the chat (n=226)		Participants who completed the chat (n=187)		Participants who started the chat (n=242)		Participants who completed the chat (n=204)		Participants who started the chat			Participants who completed the chat	
Age (y), mean (SD; range)			43.3 (9.5; 26.0 to 63.0)		43.1 (9.5; 26.0 to 63.0)		43.9 (9.6; 26.0 to 63.0)		43.5 (9.5; 26.0 to 62.0)		42.8 (9.4; 28.0 to 63.0)		42.9 (9.6; 28.0 to 63.0)		.21			.49	
**Sex, n (%)**																*.03* ^d^			*.03*
	Female	360 (76.9)		300 (76.7)		163 (72.1)		135 (72.2)		197 (81.4)		165 (80.9)							
	Male	107 (22.9)		90 (23)		62 (27.4)		51 (27.3)		45 (18.6)		39 (19.1)							
	Missing	1 (0.2)		1 (0.3)		1 (0.4)		1 (0.5)		0 (0)		0 (0)							
**Preferred language, n (%)**																.73			.69
	English	460 (98.3)		385 (98.5)		223 (98.7)		185 (98.9)		237 (97.9)		200 (98)							
	Spanish	8 (1.7)		6 (1.5)		3 (1.3)		2 (1.1)		5 (2.1)		4 (2)							
**Race and ethnicity, n (%)**																*<.001*			*<.001*
	Black	34 (7.3)		24 (6.1)		30 (13.3)		20 (10.7)		4 (1.7)		4 (2)							
	Latinx	56 (12)		48 (12.3)		25 (11.1)		21 (11.2)		31 (12.8)		27 (13.2)							
	White	323 (69)		275 (70.3)		124 (54.9)		105 (56.1)		199 (82.2)		199 (97.5)							
	Other	13 (2.8)		10 (2.6)		7 (3.1)		7 (3.7)		6 (2.5)		3 (1.5)							
	Missing	42 (9)		34 (8.7)		40 (17.7)		34 (18.2)		2 (0.8)		0 (0)							
**Urbanicity, n (%)**																*<.001*			*<.001*
	Urban	437 (93.4)		367 (93.9)		225 (99.6)		186 (99.5)		212 (87.6)		181 (88.7)							
	Rural	31 (6.6)		24 (6.1)		1 (0.4)		1 (0.5)		30 (12.4)		23 (11.3)							
Neighborhood Deprivation Index, mean (SD; range)			−0.31 (0.9; –2.17 to 1.54)		−0.32 (0.85; –2.16 to 1.54)		−0.26 (0.80; –2.17 to 1.53)		−0.32 (0.77; –2.16 to 1.38)		−0.35 (0.91; –1.74 to 1.53)		−0.32 (0.91; –1.74 to 1.54)		*.02*			*.003*	
**Algorithm criteria met, n (%)**																*<.001*			*.002*
	Colon cancer or Lynch syndrome	31 (6.6)		19 (4.9)		17 (7.5)		9 (4.8)		14 (5.8)		10 (4.9)							
	Breast cancer	83 (17.7)		67 (17.1)		35 (15.5)		27 (14.4)		48 (19.8)		40 (19.6)							
	Ovarian cancer	173 (37)		148 (37.9)		64 (28.3)		57 (30.5)		109 (45)		91 (44.6)							
	Pancreatic cancer	135 (28.8)		116 (29.7)		80 (35.4)		67 (35.8)		55 (22.7)		49 (24)							
	Multiple or other cancers	46 (9.8)		41 (10.5)		30 (13.3)		27 (14.4)		16 (6.6)		14 (6.9)							
**Number of kids, n (%)**																*<.001*			*<.001*
	0	161 (34.4)		150 (38.4)		103 (45.6)		97 (51.9)		58 (24)		53 (26)							
	≥1	250 (53.4)		241 (61.6)		95 (42)		90 (48.1)		155 (64)		151 (74)							
	Missing	57 (12.2)		0 (0)		28 (12.4)		0 (0)		29 (12)		0 (0)							
**Number of siblings, n (%)**																*<.001*			*<.001*
	0	36 (7.7)		36 (9.2)		27 (11.9)		27 (14.4)		9 (3.7)		9 (4.4)							
	≥1	375 (80.1)		355 (90.8)		171 (75.7)		160 (85.6)		204 (84.3)		195 (95.6)							
	Missing	57 (12.2)		0 (0)		28 (12.4)		0 (0)		29 (12)		0 (0)							
**Had a primary care provider, n (%)**																*.01*			.15
	Yes	382 (81.6)		318 (81.3)		195 (86.3)		158 (84.5)		187 (77.3)		160 (78.4)							
	No	86 (18.4)		73 (18.7)		31 (13.7)		29 (15.5)		55 (22.7)		44 (21.6)							

^a^NYULH: New York University Langone Health.

^b^UHealth: University of Utah Health.

^c^These *P* values compare differences between the 2 sites. The Pearson chi-square test was used for categorical independent variables, and the independent-sample *t* test was used for continuous variables (based on 2000 replicates).

^d^The italicized *P* values are less than .05.

#### Interactions With the Chatbot

Most participants initiated the chats in the afternoon (177/468, 37.1%) or evening (135/468, 30.5%). A similar trend was observed for chat end times, with most occurring in the afternoon (136/391, 35%) or evening (118/391, 30.3%). For participants who opened and started the chat (n=468), most completed it (391/468, 83.5%). Of those who completed the chat, most (315/391, 80.6%) decided to proceed with genetic testing. Of those who did not complete the chat, 74% (57/77) dropped out before any informational content. Table 3 provides detailed descriptive information on user interaction patterns.

**Table 3:** Descriptive statistics of participant chat interactions by study site.

Characteristics		Participants, n	Overall, n (%)	NYULH^a^, n (%)	UHealth^b^, n (%)	*P* value^c^
**Chat start time**		468	468 (100)	226 (48.3)	242 (51.7)	*<.001* ^d^
	Early morning (2 AM-10 AM)		53 (11.1)	10 (4.4)	43 (17.8)	
	Midday (10 AM-2 PM)		30 (6.2)	23 (10.2)	7 (2.9)	
	Afternoon (2 PM-6 PM)		177 (37.1)	117 (51.8)	60 (24.8)	
	Evening (6 PM-10 PM)		135 (30.5)	52 (23)	83 (34.3)	
	Night (10 PM-2 AM)		73 (15.1)	24 (10.6)	49 (20.2)	
**Chat end time**		391	391 (100)	187 (47.8)	204 (52.2)	*<.001*
	Early morning (2 AM-10 AM)		45 (11.6)	10 (5.4)	35 (17.2)	
	Midday (10 AM-2 PM)		29 (7.5)	21 (11.3)	8 (3.9)	
	Afternoon (2 PM-6 PM)		136 (35)	86 (46.2)	50 (24.6)	
	Evening (6 PM-10 PM)		118 (30.3)	47 (25.3)	71 (35)	
	Night (10 PM-2 AM)		61 (15.7)	22 (11.8)	39 (19.2)	
	Missing		2 (5)	1 (5.3)	1 (4.9)	
**Conversation completion status**		468	468 (100)	226 (48.3)	242 (51.7)	.71
	Completed chat		391 (83.5)	187 (82.7)	204 (84.3)	
	Did not complete chat		77 (16.5)	39 (17.3)	38 (15.7)	
**Dropout point (if the chat was not completed)**		77				.80
	Before informational content		57 (74)	28 (71.8)	29 (76.3)	
	After informational content		20 (26)	11 (28.2)	9 (23.7)	
**Total number of informational prompts selected**		391				*.02*
	0		55 (14.1)	20 (10.7)	35 (17.2)	
	1		151 (38.6)	70 (37.4)	81 (39.7)	
	2		93 (23.8)	41 (21.9)	52 (25.5)	
	≥3		92 (23.5)	56 (29.9)	36 (17.6)	
**Number of open-ended questions asked**		391				>.99
	0		350 (89.5)	167 (89.3)	183 (89.3)	
	≥1		41 (10.5)	20 (10.7)	21 (10.7)	
**Genetic testing decision**		391	391 (100)	187 (47.8)	204 (52.2)	.22
	Yes		315 (80.6)	144 (77)	171 (83.8)	
	No		8 (2)	4 (20.4)	4 (2)	
	Not sure yet		68 (17.4)	39 (20.9)	29 (14.2)	

^a^NYULH: New York University Langone Health.

^b^UHealth: University of Utah Health.

^c^These *P* values compare differences between the 2 sites, using the sample size listed on the same row for each *P* value’s calculation. The Pearson chi-square test was used for categorical independent variables, and the independent-sample *t* test was used for continuous variables with simulated *P* values (based on 2000 replicates).

^d^The italicized *P* values are less than .05.

Regarding informational prompts, more than one-third of the participants (151/391, 38.6%) opted for 1 prompt, followed by 23.8% (93/391) of users who opted for 2 prompts and 23.5% (92/391) of users who opted for ≥3 prompts. Only 14.1% (55/391) of the users opted for no informational prompts. Specifically, most of the prompts that users opted for fell under the following 4 categories: *genetics basics* (302/391, 77.2%), *lowering risk* (120/391, 30.7%), *genes included* (111/391, 28.4%), and *medical record* (110/391, 28.1%). The least opted for prompts were *gene mutations* (6/391, 1.5%), *risk percent* (4/391, 1%), and *uncertainty in risk* (0%). Except for the first prompt (*genetics basics*), which received the highest number of clicks, this likelihood of prompt selection was largely unrelated to their placement within the educational content. Regarding selecting additional cancer examples, few participants (3/391, 0.8%) chose to see the optional examples—only 0.5% (2/391) of the participants chose the ovarian cancer example, 0.8% (3/391) chose the pancreatic cancer example, and 0.5% (2/391) chose the colon cancer or Lynch syndrome example.

In terms of open-ended questions, among participants who completed the education chat (391/468, 83.5%), most (350/391, 89.5%) did not ask any questions, and 10.5% (41/391) typed at least one open-ended question for a total of 62 questions. Of the aforementioned 41 participants, 32 (78%) asked 1 question, 4 (10%) asked 2 questions, and 5 (12%) asked ≥3 questions. The most frequently asked questions were about the cost and insurance coverage of genetic testing (17/62, 27%). Other common topics were questions about logistics (16/62, 25.8%), more genetics or testing information (15/62, 24%), and personal health (9/62, 14.5%). In total, 3% (2/62) of the questions were concerned about whether genetic testing results would affect their future insurance status, and 2% (1/62) were family related. There were 7 other questions: 2 (29%) requesting a call from the service, 1 (14%) asking about coordinating the test with an upcoming blood draw, 1 (14%) seeking to change a previous answer, 2 (29%) inquiring about pausing and resuming the process, and 1 (14%) asking how privacy is protected. Table 4 provides detailed information on the open-ended questions asked by participants.

**Table 4:** Categories of open-ended questions with examples (N=62).

Nature of open-ended questions	Questions, n (%)	Examples
Financial or insurance—cost and insurance coverage	17 (27)	“Can you give me an estimate of out-of-pocket cost?” “What are the costs of your services?”
Financial or insurance—future insurance status	2 (3)	“If you test positive can it affect life insurance” “Could this affect my health insurance eligibility?”
Family	1 (2)	“Can my husband do this. Both his parents have had cancer”
Personal health	6 (10)	“Are there any health conditions that can interfere with the accuracy of the genetic test results?” “If I do have a mutation how does that change my medical care”
Logistics	15 (24)	“How long does it take to get results?” “Is this time sensitive or can it be done later”
More genetics or testing information	14 (23)	“If I test negative, what are my next steps?” “What diseases does this test cover?”
Other	7 (11)	“How is privacy protected” “Can I pause and come back later”

### Primary Outcomes

#### Number of Informational Prompts Selected

Participants had the opportunity to opt for a maximum of 9 informational prompts throughout the conversation. The odds of opting for 1 prompt, 2 prompts, and ≥3 prompts relative to 0 prompts are described in Tables 5 and 6. The models, either including race and ethnicity in the predictive block or not, indicate that only the impact of the study site was significant—participants from the NYULH site were more likely to select ≥3 informational prompts in the chat than those from the Utah site (for the model including race and ethnicity: OR 3.12, 95% CI 1.35-7.24, and *P*=.008; for the model not including race and ethnicity: OR 3.75, 95% CI 1.72-8.17, and *P*<.001). None of the other factors, including clinical and sociodemographic characteristics, were significantly associated with the number of informational prompts opted for.

**Table 5:** Multinomial logistic regression model showing associations with the number of informational prompts selected in the chat including race and ethnicity as a predictor (with 34 missing values for race and 1 missing value for sex)^a^.

Tested predictors		Total prompts selected, OR^b^ (95% CI)		
		1 prompt (n=139)	2 prompts (n=84)	≥3 prompts (n=83)
Age (y)		1.02 (0.98-1.06)	1.00 (0.96-1.05)	1.00 (0.96-1.05)
**Sex**				
	Male	—^c^	—	—
	Female	1.12 (0.47-2.64)	1.22 (0.49-3.09)	1.55 (0.60-4.00)
**Language**				
	English	—	—	—
	Spanish	N/A^d^	0.65 (0.03-13.21)	2.28 (0.18-28.09)
**Algorithm criteria met**				
	Colon cancer or Lynch syndrome	—	—	—
	Breast cancer	0.90 (0.21-3.91)	0.71 (0.14-3.58)	0.76 (0.14-3.97)
	Ovarian cancer	2.45 (0.59-10.29)	2.50 (0.54-11.67)	2.60 (0.53-12.69)
	Pancreatic cancer	2.17 (0.49-9.66)	2.39 (0.48-11.67)	2.69 (0.52-13.85)
	Multiple or other cancers	4.05 (0.67-24.63)	2.86 (0.41-19.92)	1.33 (0.17-10.48)
**Race and ethnicity**				
	Black	1.70 (0.19-15.42)	3.57 (0.39-32.65)	3.76 (0.42-33.90)
	Latinx	0.95 (0.32-2.85)	1.18 (0.38-3.71)	1.39 (0.44-4.41)
	White	—	—	—
	Other	N/A	N/A	N/A
Neighborhood Deprivation Index		1.50 (0.99-2.88)	1.20 (0.76-1.88)	1.12 (0.70-1.78)
**Had a primary care provider**				
	No	—	—	—
	Yes	1.19 (0.50-2.85)	1.29 (0.51-3.27)	1.54 (0.58-4.10)
**Study site**				
	UHealth^e^	—	—	—
	NYULH^f^	1.46 (0.67-3.19)	1.29 (0.56-2.98)	*3.12 (1.35-7.24)* ^g^
**Urbanicity**				
	Urban	—	—	—
	Rural	5.73 (0.70-46.99)	3.05 (0.33-28.11)	4.90 (0.53-45.71)
**Number of children**				
	0	—	—	—
	≥1	0.74 (0.35-1.58)	1.09 (0.48-2.46)	0.95 (0.41-2.18)
**Number of siblings**				
	0	—	—	—
	≥1	1.16 (0.32-4.20)	1.06 (0.27-4.13)	1.88 (0.44-8.04)

^a^Model statistics: χ^2^_48_=62.6, *P*=.08; Nagelkerke *R*^2^=0.17.

^b^OR: odds ratio.

^c^Reference groups in the respective models.

^d^N/A: not applicable (the limited number of cases within each category prevented the accurate retrieval of results).

^e^UHealth: University of Utah Health.

^f^NYULH: New York University Langone Health.

^g^Italicized ORs and CIs indicate statistical significance at *P*<.05; exact *P* values are reported in the main text.

**Table 6:** Multinomial logistic regression model showing associations with the number of informational prompts selected in the chat not including race and ethnicity as predictor (with 1 missing value for sex)^a^.

Tested predictors		Total prompts selected, OR^b^ (95% CI)		
		1 prompt (n=150)	2 prompts (n=93)	≥3 prompts (n=92)
Age (y)		1.02 (0.98-1.06)	1.00 (0.97-1.05)	1.00 (0.96-1.05)
**Sex**				
	Male	—^c^	—	—
	Female	0.91 (0.40-2.04)	1.16 (0.48-2.80)	1.09 (0.45-2.66)
**Language**				
	English	—	—	—
	Spanish	N/A^d^	0.61 (0.03-11.06)	2.94 (0.28-31.27)
**Algorithm criteria met**				
	Colon cancer or Lynch syndrome	—	—	—
	Breast cancer	0.93 (0.23-3.81)	0.62 (0.12-3.07)	0.67 (0.13-3.41)
	Ovarian cancer	2.20 (0.55-8.89)	2.53 (0.54-11.74)	2.83 (0.59-13.49)
	Pancreatic cancer	1.50 (0.37-6.20)	2.01 (0.42-9.53)	1.93 (0.40-9.34)
	Multiple or other cancers	2.75 (0.52-14.67)	2.58 (0.41-16.07)	1.04 (0.15-7.44)
Neighborhood Deprivation Index		1.42 (0.96-2.09)	1.32 (0.86-2.00)	1.25 (0.81-1.93)
**Had a primary care provider**				
	No	—	—	—
	Yes	1.16 (0.51-2.63)	1.32 (0.55-3.19)	1.29 (0.52-3.17)
**Study site**				
	UHealth^e^	—	—	—
	NYULH^f^	1.70 (0.84-3.47)	1.55 (0.72-3.35)	*3.75 (1.72-8.17)* ^g^
**Urbanicity**				
	Urban	—	—	—
	Rural	5.58 (0.68-45.54)	3.04 (0.33-27.89)	5.05 (0.57-46.95)
**Number of children**				
	0	—	—	—
	≥1	0.94 (0.46-1.92)	1.23 (0.57-2.64)	1.12 (0.52-2.42)
**Number of siblings**				
	0	—	—	—
	≥1	1.85 (0.66-5.20)	1.57 (0.52-4.76)	2.44 (0.75-7.90)

^a^Model statistics: χ^2^_39_=50.3, *P*=.11; Nagelkerke *R*^2^=0.13.

^b^OR: odds ratio.

^c^Reference groups in the respective models.

^d^N/A: not applicable (the limited number of cases within each category prevented the accurate retrieval of results).

^e^UHealth: University of Utah Health.

^f^NYULH: New York University Langone Health.

^g^Italicized ORs and CIs indicate statistical significance at *P*<.05; exact *P* values are reported in the main text.

#### Number of Open-Ended Questions Asked

Participants had the opportunity to ask open-ended questions to the chatbot throughout the conversation. The odds of asking one or more questions are described in the “open-ended questions” column in Table 7. The models, either including race and ethnicity as a predictor or not, indicate that none of the clinical or sociodemographic characteristics were significantly associated with whether the participant would ask open-ended questions.

**Table 7:** Logistic regression models showing predictors of chatbot use patterns (primary outcomes).

Tested predictors			Open-ended questions asked, OR^a^ (95% CI)				Genetic testing decision, OR (95% CI)		
			Models including race and ethnicity as a predictor (n=356)^b,c^		Models not including race and ethnicity as a predictor (n=390)^d,e^		Models including race and ethnicity as a predictor (n=356)^b,f^		Models not including race and ethnicity as a predictor (n=390)^d,g^
Age (y)			1.02 (0.98-1.06)		1.02 (0.98-1.05)		0.99 (0.96-1.02)		0.99 (0.96-1.02)
**Sex**									
	Male	—^h^		—		—		—	
	Female	2.40 (0.77-7.52)		2.47 (0.90-6.78)		0.80 (0.40-1.61)		0.83 (0.43-1.60)	
**Language**									
	English	—		—		—		—	
	Spanish	N/A^i^		N/A		N/A		N/A	
**Algorithm criteria met**									
	Colon cancer or Lynch syndrome	—		—		—		—	
	Breast cancer	0.82 (0.15-4.56)		0.90 (0.17-4.81)		1.54 (0.44-5.38)		1.26 (0.39-4.20)	
	Ovarian cancer	1.00 (0.21-4.91)		1.33 (0.28-6.33)		2.33 (0.72-7.53)		1.96 (0.62-6.16)	
	Pancreatic cancer	0.73 (0.15-3.85)		0.80 (0.16-4.05)		1.43 (0.44-4.66)		1.33 (0.42-4.16)	
	Multiple or other cancers	0.19 (0.02-2.29)		0.36 (0.05-2.86)		1.81 (0.46-7.16)		1.91 (0.50-7.37)	
**Race and ethnicity**									
	Black	1.18 (0.23-5.96)		—		1.24 (0.40-3.84)		—	
	Latinx	2.03 (0.77-5.33)		—		1.48 (0.59-3.68)		—	
	White	—		—		—		—	
	Other	1.88 (0.21-17.07)		—		1.04 (0.20-5.56)		—	
Neighborhood Deprivation Index			1.06 (0.69-1.65)		1.12 (0.75-1.66)		1.04 (0.73-1.46)		1.09 (0.79-1.50)
**Had a primary care provider**									
	No	—		—		—		—	
	Yes	0.83 (0.33-2.09)		0.92 (0.39-2.14)		1.07 (0.52-2.23)		1.11 (0.56-2.19)	
**Urbanicity**									
	Urban	—		—		—		—	
	Rural	1.11 (0.28-4.34)		1.15 (0.30-4.38)		0.71 (0.24-2.12)		0.76 (0.26-2.27)	
**Study site**									
	UHealth^j^	—		—		—		—	
	NYULH^k^	0.84 (0.36-1.98)		1.30 (0.63-2.70)		0.81 (0.43-1.51)		0.80 (0.45-1.42)	
**Number of children**									
	0	—		—		—		—	
	≥1	0.96 (0.42-2.20)		1.17 (0.56-2.45)		1.50 (0.82-2.75)		1.39 (0.79-2.47)	
**Number of siblings**									
	0	—		—		—		—	
	≥1	0.78 (0.21-2.92)		0.86 (0.28-2.68)		1.27 (0.47-3.45)		1.66 (0.73-3.78)	
**Total prompts**									
	0	—		—		—		—	
	1	—		—		0.66 (0.24-1.81)		0.52 (0.20-1.37)	
	2	—		—		0.60 (0.21-1.73)		0.52 (0.19-1.46)	
	≥3	—		—		0.41 (0.15-1.16)		*0.33 (0.12-0.91)* ^l^	
**Open-ended questions asked**									
	0	—		—		—		—	
	≥1	—		—		0.50 (0.22-1.16)		*0.46 (0.22-0.96)*	

^a^OR: odds ratio.

^b^34 missing values for race and 1 missing value for sex.

^c^Model statistics: χ^2^_16_=11.1, *P*=.80; Nagelkerke *R*^2^=0.07.

^d^1 missing value for sex.

^e^Model statistics: χ^2^_13_=10.3, *P*=.67; Nagelkerke *R*^2^=0.05.

^f^Model statistics: χ^2^_20_=16.1, *P*=.71; Nagelkerke *R*^2^=0.07.

^g^Model statistics: χ^2^_17_=21.1, *P*=.22; Nagelkerke *R*^2^=0.08.

^h^Reference groups in the respective models.

^i^N/A: not applicable (the limited number of cases within each category prevented the accurate retrieval of results).

^j^UHealth: University of Utah Health.

^k^NYULH: New York University Langone Health.

^l^Italicized ORs and CIs indicate statistical significance at *P*<.05; exact *P* values are reported in the main text.

#### Genetic Testing Decision

After completing the pretest genetics education chat, each participant was asked whether they would like to proceed with ordering genetic testing. The odds of a participant saying yes and requesting genetic testing are described in the “genetic testing decision” column in Table 7. The models, either including race and ethnicity in the predictive block or not, indicate that none of the clinical or sociodemographic characteristics were significantly associated with whether the participant opted to pursue genetic testing. However, user interaction was associated with the following genetic testing intention—for the model not including race and ethnicity as predictor, participants who selected ≥3 informational prompts in the chat were less likely to opt for genetic testing than those who selected no informational prompts (OR 0.33, 95% CI 0.12-0.91; *P*=.03); participants who asked one or more open-ended questions were less likely to opt for genetic testing than those who did not ask any open-ended questions (OR 0.46, 95% CI 0.22-0.96; *P*=.04). The aforementioned significant associations were not found in the model including race and ethnicity as a predictor, which had 34 missing cases.

### Secondary Outcomes

#### Conversation Completion Status

The odds of completing the pretest genetics education chat are described in the *Chatbot completion status* column in Table 8. The models, either including race and ethnicity as a predictor or not, indicate that only the impact of family cancer history was significant—in the model including race and ethnicity as a predictor, participants with family history of ovarian cancer (OR 3.26, 95% CI 1.30-8.20; *P*=.01), pancreatic cancer (OR 3.27, 95% CI 1.26-8.47; *P*=.02), and multiple or other cancers (OR 4.22, 95% CI 1.22-14.58; *P*=.02) were more likely to complete the chat than those with family history of colon cancer or Lynch syndrome. In the model not including race and ethnicity as a predictor, participants with family history of breast cancer (OR 2.66, 95% CI 1.06-6.70; *P*=.04), ovarian cancer (OR 3.72, 95% CI 1.58-8.76; *P*=.003), pancreatic cancer (OR 3.90, 95% CI 1.62-9.41; *P*=.002), and multiple or other cancers (OR 5.43, 95% CI 1.65-17.84; *P*=.005) were more likely to complete the chat than those with family history of colon cancer or Lynch syndrome. None of the other clinical or sociodemographic characteristics significantly predicted whether the participants would complete the chat.

**Table 8:** Logistic regression models showing associations with chatbot use patterns (secondary outcomes).

Tested predictors			Conversation completion status, OR^a^ (95% CI)				Dropout point, OR (95% CI)		
			Model including race and ethnicity as a predictor (n=425)^b^^,c^		Model not including race and ethnicity as a predictor (n=467)^d^^,e^		Model including race and ethnicity as a predictor (n=69)^f^^,g^		Model not including race and ethnicity as a predictor (n=77)^h^^,i^
Age (y)			1.00 (0.97-1.03)		0.99 (0.97-1.02)		1.00 (0.93-1.08)		1.00 (0.93-1.07)
**Sex**									
	Male	—^j^		—		—		—	
	Female	1.04 (0.53-2.05)		0.96 (0.51-1.81)		1.76 (0.37-8.32)		1.05 (0.25-4.35)	
**Language**									
	English	—		—		—		—	
	Spanish	0.79 (0.08-7.66)		0.59 (0.11-3.07)		N/A^k^		3.87 (0.15-97.67)	
**Algorithm criteria met**									
	Colon cancer or Lynch syndrome	—		—		—		—	
	Breast cancer	2.16 (0.80-5.79)		*2.66 (1.06-6.70)* ^l^		0.31 (0.03-2.99)		0.39 (0.05-3.16)	
	Ovarian cancer	*3.26 (1.30-8.19)*		*3.72 (1.58-8.76)*		0.89 (0.14-5.80)		1.36 (0.24-7.79)	
	Pancreatic cancer	*3.27 (1.26-8.47)*		*3.90 (1.62-9.41)*		1.93 (0.31-12.10)		2.17 (0.40-11.67)	
	Multiple or other cancers	*4.22 (1.22-14.58)*		*5.43 (1.65-17.84)*		0.67 (0.04-10.92)		0.83 (0.06-11.97)	
**Race and ethnicity**									
	Black	0.50 (0.19-1.27)		—		0.95 (0.14-6.58)		—	
	Latinx	1.14 (0.47-2.72)		—		2.49 (0.36-17.34)		—	
	White	—		—		—		—	
	Other	0.52 (0.13-2.03)		—		1.51 (0.10-23.60)		—	
Neighborhood Deprivation Index			0.94 (0.68-1.31)		0.92 (0.69-1.23)		1.07 (0.52-2.19)		1.41 (0.75-2.64)
**Had a primary care provider**									
	No	—		—		—		—	
	Yes	0.85 (0.41-1.78)		1.00 (0.51-1.97)		0.46 (0.08-2.54)		0.85 (0.18-4.12)	
**Urbanicity**									
	Urban	—		—		—		—	
	Rural	0.52 (0.20-1.37)		0.56 (0.22-1.43)		1.62 (0.16-16.03)		1.28 (0.17-9.72)	
**Study site**									
	UHealth^m^	—		—		—		—	
	NYULH^n^	0.86 (0.47-1.57)		0.92 (0.69-1.23)		0.94 (0.10-8.61)		1.24 (0.35-4.32)	

^a^OR: odds ratio.

^b^42 missing values for race and ethnicity and 1 missing value for sex.

^c^Model statistics: χ^2^_14_=15.0, *P*=.38; Nagelkerke *R*^2^=0.06.

^d^1 missing value for sex.

^e^Model statistics: χ^2^_11_=14.3, *P*=.22; Nagelkerke *R*^2^=0.05.

^f^8 missing values for race and ethnicity and 1 missing value for sex.

^g^Model statistics: χ^2^_14_=6.8, *P*=.94; Nagelkerke *R*^2^=0.14.

^h^No missing values.

^i^Model statistics: χ^2^_11_=5.6, *P*=.90; Nagelkerke *R*^2^=0.10.

^j^Reference groups in the respective models.

^k^N/A: not applicable (the limited number of cases within each category prevented the accurate retrieval of results).

^l^Italicized ORs and CIs indicate statistical significance at *P*<.05; exact *P* values are reported in the main text.

^m^UHealth: University of Utah Health.

^n^NYULH: New York University Langone Health.

#### Dropout Point

For participants who did not complete the chat conversation, they could have dropped out of the chat before viewing any informational content or after starting to interact with informational content. The odds of dropping out of the chat after the informational content are described in the “dropout point” column in Table 8. The models, either including or excluding race and ethnicity as a predictor, indicate that none of the clinical or sociodemographic characteristics significantly predicted whether the participant would drop out of the chat after beginning to view any informational content. However, it is important to note that the vast majority did drop out before viewing informational content.

## Discussion

### Principal Findings

Many patients have limited access to genetic services, and due to the increasing need to deliver cancer genetic services for improved health outcomes, alternative delivery models for pretest genetics education are gaining more attention [3]. Recently, researchers have been exploring how chatbots can be used as a cost-effective model for such delivery [25]. By taking on routine educational tasks, these automated tools help address the staffing bottleneck that genetic service providers often face, ultimately improving patient access to critical services and enhancing overall health outcomes [1]. However, limited attention has been paid to how users actually interact with these chatbots and how their sociodemographic backgrounds may influence these use patterns. This study explored user interaction patterns with our chatbot, examined how clinical and sociodemographic factors influence these patterns, and assessed how user characteristics and interaction behaviors affect decisions regarding genetic testing. In summary, our findings highlighted the chatbot’s acceptability to users—of the users who started the chat, most completed it and opted for additional information. In addition, participants demonstrated a strong willingness to undergo genetic testing. We found that most sociodemographic factors did not significantly influence user interaction patterns. This potentially indicates the chatbot’s scalability across diverse populations if they can access the chat. Future efforts could explore other potential predictors such as health literacy levels [53] and individual dispositional traits such as tolerance for uncertainty [54]. Importantly, participants with high informational needs (ie, those who selected ≥3 informational prompts or asked at least one open-ended question) were less likely to opt for genetic testing. This finding highlights the importance of further enhancing and personalizing the chatbot’s educational content to meet users’ informational needs, as well as the potential value of offering follow-up consultations with genetic experts to address remaining questions and concerns in clinical practice.

This study closely investigated how users interacted with the chatbot, focusing on whether they tended to seek additional information within the conversation and ask further open-ended questions. Regarding how users interacted with the informational prompts and relevant examples, we have 3 main findings. First, although we found that the vast majority of users selected at least one prompt, a closer examination revealed differences in the selection of individual prompts. Specifically, 4 prompts received a high number of clicks, whereas others, such as *gene mutations*, *risk percent*, and *uncertainty in risk*, were rarely selected. We discovered that the likelihood of a prompt being selected was largely unrelated to its placement within the educational content. Future research should aim to understand why users select particular prompts and explore ways to enhance the appeal of prompts, such as by improving prompt title framing, images, or page descriptions [55]. Second, we observed that users from the 2 different sites varied in the number of prompts selected. Participants from the 2 sites differed not only in their selection of informational prompts but also in various user characteristics and interaction details. These findings suggest a need to further explore differences in information needs among various patient populations and develop ways to tailor educational content and delivery formats to meet these needs. In addition, only a very small number of users chose to view the 3 optional examples of genetic risk. Considering the power of exemplars in information comprehension and various persuasion contexts [56], future research should focus on increasing users’ motivation to view examples to enhance informed decision-making. This could be achieved by enhancing the effectiveness and attractiveness of the first example viewed to increase users’ interest in viewing more or by personalizing example recommendations based on users’ family cancer history.

Our examination of how users engaged with open-ended question features can provide valuable insights into enhancing the usability of cancer genetic service chatbots. Low response rates and poor quality of answers to open-ended questions have consistently posed challenges in user surveys [57]. Our study revealed that only a very small number of users posed at least one open-ended question (especially knowledge related). This might suggest that the scripted educational content adequately addressed their information needs. However, it is important to note that open-ended questions generally require more cognitive effort from users than closed-ended questions and scripted educational content [57,58]. Future research could focus on facilitating user engagement with open-ended questions, such as incorporating voice input options [59]. In addition, evaluating chatbot usability could include direct inquiries after chat completion to assess whether users feel that their information needs are fully met. Regarding the types of open-ended questions asked, a significant portion related to financial and insurance matters. In contrast to the previous feasibility study, in which users primarily asked about whether and what portion of testing costs would be covered by insurance [33], in this study, users were also interested in whether their genetic test results might impact their future health insurance status. Future genetic education initiatives could provide additional pertinent information on this subject, helping alleviate user concerns regarding genetic testing results, such as perceived stigma and insurance discrimination [60].

We further delved into the specifics of use patterns. First, the high chat completion rate indicates the acceptability of the chatbot design and user overall satisfaction. Users with different cancer family histories showed differences in their likelihood of completing the conversation. This insight highlights the importance of making further efforts to provide more targeted information that addresses the needs of individuals with different cancer family histories, as well as personalizing educational content based on their specific clinical contexts. Tailoring content to match user characteristics may enhance patient engagement with educational materials [33]. Second, of the small number of users who exited the chat before completion, a substantial majority left before encountering any informational content. For those who dropped out of chatbot services, as stated previously, the team followed up via multiple channels (ie, phone calls, emails, and mailed letters) to answer any questions and provide additional information. Future efforts may focus on examining whether the chatbot format or interface discourages users from continuing the chat. Follow-up interviews or surveys with these participants could help identify specific reasons for dropout and provide valuable suggestions for improving chatbot design and features to better meet users’ needs.

Notably, although the chatbot service delivery model performed equivalently to the standard-of-care model in providing pretest cancer genetic services and promoting genetic testing, most patients invited and assigned to either model chose not to initiate the pretest genetic services [29]. While this is consistent with other studies of pretest services [61,62], future efforts may focus on strategies to encourage potential patients to initiate a chat or participate in any type of cancer genetic pretest services. Previous findings from the BRIDGE trial have shown that engagement with pretest genetic services under both service delivery models is positively associated with previous health care interactions, including frequent use of the patient portal and primary care visits [63]. These findings suggest that promoting regular use of patient portals through improved usability and more user-friendly digital health navigators may help expand the reach of genetic services [63,64]. Increasing access to primary care, building trust in the health care system, and enhancing overall health care accessibility may also promote greater engagement with genetic services [63,65,66]. In addition, it is essential to develop strategies that enhance individuals’ motivation to act on their genetic cancer risks and incorporate these risks into their overall health care considerations [67,68]. The public’s generally low level of knowledge about genetic cancer, combined with the absence of immediate symptoms, may lead to underestimation of these risks and reduced perceived value of pretest services [69,70]. To address this, future efforts should place greater emphasis on informal and broader public education delivered through health care providers and media platforms, especially by promoting foundational knowledge and awareness of cancer genetics [71,72]. Enhancing public understanding of these risks may, in turn, increase individuals’ interest in and motivation to engage with genetic pretest services [69,73].

The ultimate goals of developing alternative pretest education models, such as using chatbots, are to enhance patients’ comprehensive knowledge of cancer genetics and support their related decision-making processes [29]. Therefore, another core objective is to understand participants’ decisions regarding genetic testing following the chat. Most users expressed a willingness to undergo genetic testing after completing the pretest education content. Specifically, we did not find any clinical or sociodemographic factors that influenced their decision to pursue genetic testing. However, we observed that users with a very high level of engagement with the chatbot, such as those who opted for ≥3 informational prompts or asked open-ended questions, had a lower likelihood of undergoing genetic testing. Consistent with the findings from the feasibility study, we believe that this finding indicates that, when individuals have a strong informational motivation regarding a health issue—seeking more information and having more questions—they may find it more challenging to make an immediate decision regarding the health issue [33,74]. Recognizing that individuals with high information needs may not immediately pursue genetic testing, it is essential to meet their informational needs over time to support their decision-making process.

Precision medicine emphasizes the importance of enabling informed health decisions, helping individuals understand their personal health status, the available options, and the potential benefits and consequences of each choice [75]. This aligns with our original intention to enhance the public’s informed decision-making on cancer genetic issues through diverse and accessible delivery models [29]. Future chatbot development should focus on continuously improving the answer pool for user-submitted open-ended questions. Common concerns and frequently asked questions should be proactively integrated into the core educational content. Beyond enhancing the chatbot’s information delivery, it is also important to consider how human support can complement the service. Previous research has suggested combining chatbot education with human counseling to deliver cancer pretest genetic services [16]. Therefore, in clinical practice, follow-up mechanisms could be implemented where genetic counseling assistants respond to users whose open-ended questions were not fully addressed by the chatbot, similar to the procedures in our trial. For users who interacted with numerous informational prompts but did not proceed with testing, follow-up from the genetic counseling team may help identify specific concerns and provide additional guidance and emotional support. Moreover, it is essential to gain a deeper understanding of the characteristics of users with higher information needs. Although our current models did not identify significant sociodemographic or clinical predictors, future research could explore other relevant factors, such as personal or broader family health history beyond cancer. Health literacy and genetics-specific literacy may also influence the amount of information that users seek before making testing decisions [76,77]. In addition, psychological traits such as intolerance of uncertainty (the degree to which individuals find uncertainty stressful or difficult to tolerate) [54] may influence their desire for more information. By identifying these characteristics, we can design cancer pretest genetic services that offer more tailored, relevant, and personalized information to better support individuals with high information needs.

As new technologies, particularly generative artificial intelligence (AI), continue to advance, there is a growing interest in harnessing these innovations to enhance chatbot health service delivery. AI-driven or machine learning–based chatbots provide personalized and engaging interactions tailored to the unique needs of users [78]. However, concerns persist regarding the accuracy and unpredictability of the information they deliver, violation of scope boundaries (eg, providing health care advice outside the intended scope of a chatbot), and the potential violation of user privacy [79,80]. In contrast, traditional rule-based chatbots use scripted content created by expert research teams. In our case, the scripted material was derived from actual consultations during pretest cancer genetic counseling sessions. While this method ensures a high level of professional accuracy and consistency, it may lack sufficient personalization [81]. Future efforts may combine the strengths of both rule-based and AI-driven content to optimize user satisfaction and engagement with pretest genetic education.

In the field of cancer genetic testing, theoretical frameworks are still underused [82]. To enhance chatbot interactivity in a theory-driven manner, we recommend focusing on the following. First, drawing from the technology acceptance model, increasing user engagement with new technology (or services based on new technologies) requires enhancing both perceived usefulness and ease of use [83]. As advancements in IT and AI continue, it is critical to make chatbot content more informative and user-friendly to enhance user engagement and intention. Second, the theory of motivated information management highlights how individuals manage information-seeking behavior [84]. This process involves 3 stages: the interpretation phase (recognizing uncertainty or anxiety about an issue), the evaluation phase (weighing the benefits of seeking information and personal capacity to do so), and the decision phase (choosing how to manage the information search) [84]. To design better chatbots for cancer genetics, it is essential to identify the primary concerns of users and their key uncertainties and incorporate design features that enhance their confidence and motivation to engage with the chatbot. In summary, future cancer genetic chatbot designs should leverage insights from emerging theory-based literature on public acceptance of new technology while carefully considering the unique aspects of cancer genetic information and user motivations to seek it. This approach will lead to more effective and user-centered chatbot cancer genetic services.

### Strengths and Limitations

Compared with the small feasibility study by Chavez-Yenter et al [33], the data from the full trial significantly expanded the sample size, study sites, and diversity in terms of race and ethnicity. This study conducted a more detailed examination of user interaction and use patterns in a real-world health care setting at 2 different sites, each with patients with distinct sociodemographic characteristics. However, several limitations need to be acknowledged. First, there were several technical issues with the commercial platform. As noted previously, we were unable to download 13 user interaction transcripts for log analysis. While this represents a small data loss (13/468, 2.8% of the participants), which likely did not impact the overall patterns of our findings, it nonetheless reduced the completeness of our dataset. In addition, the commercial platform used in this study only provided users’ session start and stop times but did not capture pause durations. This hindered our ability to calculate users’ “time on task,” which we believe is an important descriptive metric worth reporting and analyzing in future studies. Second, this trial offered the chatbot through patient portal messages (MyChart). While this approach was convenient and scalable, it may have introduced selection bias by primarily reaching individuals who are already digitally engaged—those with access to the portal, sufficient digital health literacy, and reliable internet access. Thus, this method may have excluded individuals whom the health care system has long underserved. Although we had a much larger and more diverse participant pool than the earlier feasibility trial, the participants in this trial were still predominantly non-Hispanic White and female individuals. Moreover, the number of Spanish-preferred users and rural participants was also very low. These demographics may reflect a bias in user composition due to disparities in access to digital health resources, such as the patient portal, and the presence of family history documentation within the EHR [85]. Accordingly, future efforts could be directed in the following ways. To improve equitable access to genetic pretest services, we recommend implementing additional outreach strategies to enhance the collection of information on family history of cancer. To alleviate the burden of digital literacy on chatbot engagement, we propose implementing and testing additional features, including AI-powered interactions and voice input options for submitting open-ended questions. These additional features may help mitigate the negative impact of limited digital literacy or experience on user interactions with the chatbot. For individuals without home internet or access to digital health tools, digital health approaches can be supplemented with in-person and telehealth approaches.

Third, our findings may be limited by incomplete or biased family history data in the EHR. Our inclusion criteria were based on the 2018 National Comprehensive Cancer Network guidelines. While the review of family history information by genetic counseling assistants ensured that those who receive testing met the criteria, our prior work has shown systematic underidentification due to biases in the available cancer family history information in the EHR [85]. This also highlights the importance of enhancing the collection of family history information within the health care system. Fourth, we may need to make additional efforts to capture users’ chatbot interaction experiences more effectively and identify more nuanced characteristics that influence their engagement patterns. In this study, we focused solely on observed and recorded interaction patterns as our main focus was to track and understand how users interacted with the chatbot. Future studies could include postinteraction surveys after the chat to assess user experience, such as their perceived satisfaction and helpfulness of the chatbot, offering a more comprehensive understanding of the overall interaction experience. Meanwhile, although we aimed to explore the association between clinical and sociodemographic factors and use patterns, we did not find many significant predictors. This may suggest some scalability of the chatbot content and features. However, this may also be because the factors we included were insufficient to capture some important and relevant characteristics. Future studies should explore additional clinical and sociodemographic factors such as general personal disease history and family disease history, medical mistrust toward health care systems, and perceived social support networks, as well as other potential predictors, including literacy levels and motivations for seeking information specific to cancer genetics.

### Conclusions

Despite the limitations, the results of this large-scale trial demonstrate that chatbots can serve as a cost-efficient, convenient, and broadly applicable alternative for pretest cancer genetic education. Our analysis of user interaction patterns with the chatbot content revealed its effectiveness in engaging users, as indicated by the high completion rate and frequent selection of informational prompts. In addition, we examined how clinical and sociodemographic factors impact these interaction patterns, providing insights to improve the scalability of chatbot services across diverse population groups and support personalization to better address the needs of individuals from different backgrounds. Enhancing individuals’ genetic knowledge and cognitive skills enables them to make informed decisions about genetic service uptake, such as genetic testing [86]. By outlining user interaction patterns with cancer genetic education chatbots, our findings offer further empirical support for chatbots as an effective delivery model, potentially accelerating the enhancement of genetic literacy among the broader public and contributing to the prevention of cancer-related genetic diseases.

## Appendix

Multimedia Appendix 1 Scripts for the Broadening the Reach, Impact, and Delivery of Genetic Services pretest genetics education chatbot.

## Abbreviations

AI: artificial intelligence
BRIDGE: Broadening the Reach, Impact, and Delivery of Genetic Services
EHR: electronic health record
NYULH: New York University Langone Health
OR: odds ratio
REDCap: Research Electronic Data Capture
UHealth: University of Utah Health

## Data Availability

The datasets generated or analyzed during this study are available from the corresponding author on reasonable request.
