# Supplementary material for: Bridging Technology and Pretest Genetic Services: Quantitative Study of Chatbot Interaction Patterns, User Characteristics, and Genetic Testing Decisions
Source: J Med Internet Res. 2025 Sep 17;27:e73391. doi: 10.2196/73391 (PMC12489413; doi:10.2196/73391)
Supplement: Multimedia Appendix 1 [file jmir_v27i1e73391_app1.pdf]

**Patient receives this pretest education chat if identified by BRIDGE as meeting criteria for testing.**
Gia: Hi, {patientName}.<br><br>Gia: I'm GIA, your automated Genetic Information Assistant.<br><br>Gia: On behalf of the University of Utah healthcare team, I'm reaching out to patients who might benefit from genetic testing.<br><br>Patient: Hi Gia<br><br>Gia: Before we get started, here's a quick message from Wendy at University of Utah genetics.<br><br>*** INTRO VIDEO ***<br><br>Patient: Let's continue<br><br>Gia: Based on the family health history in your medical record, it looks like you meet criteria for testing of genes related to certain cancers.<br><br>Gia: The healthcare team at University of Utah recommends having this genetic testing to provide more information about your cancer risk and to help plan your care.<br><br>Gia: It can also be important for your family members, including children if you have them.<br><br>Patient: I see<br><br>Gia: In this chat I'll ask you some basic information about your family, and provide information about why testing is important, how it is done, and what results are possible.<br><br>Gia: At the end, I can answer any additional questions you have.<br><br>Patient: Sounds good<br><br>Gia: At any time during our conversation you can click the menu button (☰) to access additional options such as increasing font size or read speed.<br><br>Gia: You can also click on any of your responses to change them.<br><br>Gia: When we're done chatting, a team member will contact you to help with next steps.

Patient: Let's continue	Patient: I'll come back later<br><br>Gia: Ok, when you come back we'll continue where we left off.<br><br>Gia: The sooner you complete our chat, the sooner we can take next steps.<br><br>Patient: I'm ready to continue now

Gia: Great!

Gia: Let's start with the questions about your family.

Gia: I know everyone's family is different and that a blood relationship is not always what defines family.

Gia: But because we're talking about genetics, I will only be asking for information about your biological (or blood) relatives.

Gia: First, do you have any siblings?

Patient: Yes / No / I'm not sure

Gia: How many brothers do you have? (includes full and half brothers)

Patient: *Types in*

Gia: How many sisters do you have? (includes full and half sisters)

Patient: *Types in*

Gia: And do you have any children?

Patient: Yes / No

Gia: How many sons do you have?

Patient: Chooses from options 0 - 10

Gia: How many daughters do you have?

Patient: Chooses from options 0 - 10

Gia: Thank you for answering those questions.

Gia: Now let's review some basics of genetics and cancer.

Patient: Sounds good

Gia: Here's a diagram that I think is helpful.

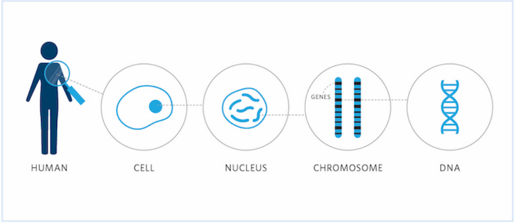

Gia: Your body is made up of trillions of cells, like the one in the diagram and each cell contains structures called chromosomes.

Gia: Chromosomes come in pairs, represented by the 2 lines, one from your mother and one from your father.

Gia: The chromosomes contain genes and each gene does a specific job such as controlling hair or eye color for example.

Gia: Sometimes a gene has a change in the genetic code. A change that keeps a gene from doing its job correctly is called a mutation.

Patient: I see / This is sounding familiar now

Gia: Some genes have the job of preventing cancer.

Gia: If a person has a mutation in a gene that is supposed to be preventing cancer, then their risk of cancer can be increased.

Patient: I have a good understanding of genetics

Gia: Great. As you may know, your cells contain chromosomes which come in pairs.

Gia: The chromosomes contain genes and each gene does a specific job such as controlling hair or eye color.

Gia: Sometimes a gene has a change in the genetic code. A change that keeps a gene from doing its job correctly is called a mutation.

Gia: If a person has a mutation in a gene whose job is to prevent cancer then their risk of cancer can be increased.

Gia: It doesn't mean that a person will definitely get cancer and their degree of risk depends on many factors.

Patient: Ok / Yes, we're on the same page

<table>
<tr><td>Gia: It doesn’t mean that person will definitely get cancer and their degree of risk depends on many factors.

Patient: Ok / Interesting!</td><td></td></tr>
<tr><td colspan="2">Gia: Cancer can be inherited, or it can occur randomly.

Gia: Cancer that is caused by a gene mutation is “hereditary”, meaning that it is passed down within families.

Gia: Other cancer is random, or “sporadic,” which means that it’s caused by things like aging, lifestyle, and environment.</td></tr>
<tr><td>Patient: I understand</td><td>Patient: Can you explain this in another way?

Gia: Yes! Here's a short video that explains it nicely.

[Video]

Patient: Thanks, that was helpful / Let’s continue</td></tr>
<tr><td colspan="2">Gia: Everyone has some level of cancer risk. Most people have what is known as "average" or "population" risk.

Gia: People with a close family member with cancer, such as a parent or sibling, generally have an increased risk for developing that type of cancer.

Gia: Those that test positive for a gene mutation related to cancer may have an even higher risk.</td></tr>
<tr><td>Patient: I see</td><td>Patient: Can you give me an example?

Gia: Of course!

Gia: Here's an illustration of lifetime breast cancer risk for a woman. ***IMAGE***

Gia: The average lifetime risk of breast cancer is 10%.</td></tr>
</table>

<table>
<tr><td rowspan="3"></td><td colspan="4">Gia: Having a mother or sister with breast cancer increases the risk to 15 - 25%.

Gia: And those with gene mutations in the gene called BRCA1 have a 50 - 70% risk for breast cancer.</td></tr>
<tr><td rowspan="2">Patient: Thanks, that helps</td><td colspan="2">Patient: Why is there a range of percentages?

Gia: The exact risk depends on many factors including the specific gene mutation, if there is one, and the details of the family history.

Gia: You can talk to a genetic counselor to help you understand your personal risk.</td><td rowspan="2">Patient: Can I see more examples?

Loop through offering other examples below</td></tr>
<tr><td>Patient: Ok</td><td>Patient: Can I see more examples?

Loop through offering other examples below</td></tr>
</table>

Ovarian cancer

Gia: Here's an illustration of lifetime ovarian cancer risk for a woman. ***IMAGE***

Gia: The average lifetime risk of ovarian cancer is 1%.

Gia: Those with family history have a risk of 3 - 5%.

Gia: And those with a high-risk gene mutation have a risk of 20 - 50%.

Colon cancer

Gia: Here's an illustration of lifetime colon cancer risk. ***IMAGE***

Gia: The average lifetime risk of colon cancer is 5%.

Gia: Those with family history have a risk of 8 - 10%.

Gia: And those with a high-risk gene mutation have a risk of 20 - 50%.

Pancreatic cancer

Gia: Here's an illustration of lifetime pancreatic cancer risk. ***IMAGE***

Gia: The average lifetime risk of pancreatic cancer is 1%.

Gia: Those with family history have a risk of 2 - 3%.

Gia: And those with a high-risk gene mutation have a risk of 5 - 20%.

Gia: There are specific things we look for in a family's cancer history to figure out whether the cancer is likely to be hereditary.

Gia: You may have been identified as eligible for genetic testing because we found one or more of these things in your medical record.

Patient: What kind of things do you look for?<br><br>Gia: Some of the things we look for are:<br><br>Gia: Having cancer at a young age (usually younger than 50)<br><br>Gia: Having multiple family members with cancers that are known to share a common genetic link<br><br>Gia: Some rare cancer types such as ovarian cancer, men with breast cancer, or pancreatic cancer	Patient: Ok, Gia

<table>
<tr><td>Gia: Jewish ancestry in combination with certain cancers

Patient: Got it</td><td></td></tr>
<tr><td colspan="2">Gia: The genetic testing we are offering you looks at many genes that are related to hereditary cancer.

Gia: Each gene is unique in terms of which cancer(s) it relates to, the level of risk it causes, and how it impacts care.</td></tr>
<tr><td>Patient: I see</td><td>Patient: Which genes are included?

Gia: We test for mutations in genes you may have heard of like BRCA1 and BRCA2.

Gia: And also, for many other genes that you likely are not familiar with.

Gia: If you have questions about specific genes, we can answer those when we talk to you on the phone.

Patient: Got it</td></tr>
<tr><td colspan="2">Gia: If we find a mutation in one of these genes, additional or more frequent cancer screening may be recommended to help detect cancer early.

Gia: For some types of cancer there might be other options that can help lower cancer risk.

Patient: Ok / What kind of options? / Do I have to get tested?</td></tr>
</table>

<table>
<tr>
<td>Patient: Do I have to get tested?

Gia: Testing is your choice, but this information could help your healthcare team plan what cancer screening is best for you.

Gia: If you decide to get tested, you will be well supported.

Gia: If you decide not to get genetic testing, we can only give recommendations for cancer screening based on your personal and family history.

Gia: At the end of this chat you can decide if you want testing and can talk to a genetics professional to get more information.

Patient: Ok</td>
<td>Patient: What kind of options?

Gia: It depends on your specific situation.

Gia: For example, if you are at high risk of developing breast cancer, you may be offered a mammogram and breast MRI every year to screen you more closely.

Gia: Surgical options may be considered with some mutations.

Patient: Ok / Do I have to have testing?</td>
</tr>
</table>

Gia: There are 3 types of results that we may find when we look at your genes. Let me describe them for you:

1) **Positive**: A gene mutation was found that increases the chances of developing certain cancers. Laboratories may call a mutation a "pathogenic variant" on a test report.

2) **Negative**: No gene mutations that increase the chance of developing cancer were found in any of the genes tested.

3) **Variant of Unknown Significance (VUS)**: A variation was found in one of your genes, but the variation has not been proven to increase the risk of cancer. This is not a positive result and should not be used to change your medical management.

Patient: More on positive result	Patient: More on negative result	Patient: More on VUS result	Patient: Let's move on

Gia: If you have a positive result, your doctor or a genetic counselor will walk you through the next steps.<br><br>Gia: It's important to know that if you test positive, it does not mean you will definitely develop cancer.	Gia: A negative result would help reassure you that you do not have a gene mutation causing a higher risk for cancer.<br><br>Gia: However, you may still have an increased risk for cancer based on your family history.<br><br>Gia: The genetic counseling team can help a relative have genetic testing.	Gia: All people have variations in their genes. This is part of what makes each person unique.<br><br>Gia: Sometimes genetic testing identifies variations and we can't say for sure yet if they are linked to cancer.<br><br>Gia: As we learn more, most of these variants are found to be normal (not associated with cancer or other disease).<br><br>Gia: If we find you have a VUS we will keep track of it and notify you when we learn more about it.<br><br>Patient: Got it / 👍<br><br>Gia: Even with a VUS result, you may still have an increased risk for cancer based on your family history.<br><br>Gia: A genetic counselor will review your family history and let you know what cancer screening is recommended.	

<table>
<tr>
<td rowspan="2">Patient: Let’s move on / Ok</td>
<td>Patient: Let’s move on / Ok</td>
<td></td>
</tr>
<tr>
<td colspan="2">If didn’t already cover more info on positive result (otherwise skip this section)

Gia: Sounds good

Gia: If you have a positive result, your doctor or a genetic counselor will walk you through the next steps.

Gia: It’s important to know that if you test positive, it does not mean you will definitely develop cancer.

Patient: Ok</td>
</tr>
<tr>
<td colspan="3">Gia: If you are found to have a mutation, it will be important to share this information with family members.

Gia: Close relatives like children, siblings and parents have a 1 in 2 (or 50%) chance of having the same mutation and other relatives may also have increased risk.

Gia: If needed, we can help your relatives get testing.</td>
</tr>
<tr>
<td colspan="3">Patient: Got it / How is privacy protected?

Gia: Only you and your healthcare providers will have access to your results.

Gia: Your report will go into your medical record so those involved in your care can review it as needed.

Gia: It is up to you whether you choose to share your genetic test results with others outside of the healthcare system.

Gia: However, as we have discussed it is often very helpful to share your genetic test results with family members.</td>
</tr>
</table>

Patient: Ok

Gia: Also, you are protected by GINA, the Genetic Information Nondiscrimination Act.

Gia: GINA provides specific protections related to health insurance and employment. For more complete information click **here**.

Patient: Ok / Thank you

Gia: If you're interested in genetic testing for cancer risk, the genetics team will give you a call to discuss next steps.

Gia: We'll work with you on insurance coverage. Out of pocket cost is usually $100 or less.

Gia: Before I ask if you're interested in testing, do you have any additional questions?

Clear AI

Gia: What do you think? Would you like to move forward with testing?

Patient: Yes<br><br>Gia: Great. When we call you, we'll go over instructions for providing a sample for the testing and to answer any additional questions about insurance.<br><br>Gia: A kit can be sent to your home in the next 72 hours, and results will be available in 3 to 4 weeks after you send the sample.<br><br>Gia: Here's some more information on next steps from Wendy at	Patient: No<br><br>Gia: Ok. Someone on the team might reach out to you to make sure you got all the information you need.	Patient: I'm not sure yet<br><br>Gia: Ok, I'll pass that information on to the team.<br><br>Gia: Someone will reach out to you about your questions.

University of Utah genetics.		
Gia: Thank you {patient name} for taking the time to chat with me.<br><br>Gia: Have a great day. Goodbye!<br><br>*end chat*		

This is a Multimedia Appendix to a full manuscript published in the J Med Internet Res. For full copyright and citation information see http://dx.doi.org/10.2196/jmir.73391
